# Detection of Alzheimer Neuropathology in Alzheimer and Non-Alzheimer Clinical Syndromes With Blood-Based Biomarkers

**DOI:** 10.1001/jamaneurol.2024.5017

**Published:** 2025-02-10

**Authors:** Lawren VandeVrede, Hanna Cho, Mark Sanderson-Cimino, Fattin Wekselman, Yann Cobigo, Maria Luisa Gorno-Tempini, Hilary W. Heuer, Joel H. Kramer, Argentina Lario Lago, Dana Leichter, Peter Ljubenkov, Bruce L. Miller, David C. Perry, Gil D. Rabinovici, Julio C. Rojas, Howard J. Rosen, Rowan Saloner, Adam Staffaroni, Gallen Triana-Baltzer, Salvatore Spina, William W. Seeley, Lea T. Grinberg, Hartmuth C. Kolb, Renaud La Joie, Adam L. Boxer

**Affiliations:** 1 Memory and Aging Center, UCSF Weill Institute for Neurosciences, University of California, San Francisco; 2 Department of Neurology, Gangnam Severance Hospital, Yonsei University College of Medicine, Seoul, Republic of Korea; 3 Associate Editor, *JAMA Neurology*; 4 Neuroscience Biomarkers, Janssen Research & Development, San Diego, California; 5 Department of Pathology, University of California, San Francisco

## Abstract

**Importance:**

Blood-based biomarkers for Alzheimer disease (AD) are clinically available, but their value is not well understood in syndromes typically associated with frontotemporal lobar degeneration syndromes (FTLD).

**Objective:**

To investigate the clinical importance and detectability of AD in FTLD-related neurodegenerative syndromes using 3 plasma biomarkers, phosphorylated tau 217 (p-tau217), neurofilament light chain (NfL), and glial fibrillary acidic protein (GFAP).

**Design, Setting, and Participants:**

This clinicopathological study took place at the University of California San Francisco Alzheimer Disease Research Center from August 2008 to July 2022. Autopsied individuals with clinical evaluation and neuropathological examination, diagnosed with clinical syndromes related to AD (n = 125), frontotemporal lobar degeneration (FTLD; n = 198), or cognitively unimpaired (CU) at the time of evaluation (n = 16) were included.

**Exposures:**

AD-related or FTLD-related clinical syndromes or CU.

**Main Outcomes and Measures:**

P-tau217, NfL, and GFAP were measured with single-molecule array (SIMOA). AD was defined as intermediate or high AD neuropathological change (ADNC) at autopsy. Clinical biomarker associations were evaluated using linear regressions. Imaging analyses used bayesian linear mixed-effects modeling.

**Results:**

A total of 349 individuals (191 [55%] male; mean [SD] age at death, 72 [11] years) were included. AD was common in both AD-related syndromes (110/125 [88%]) and FTLD-related syndromes (45/198 [23%]). Neuropathological stage at autopsy was higher in AD-related syndromes (high ADNC: 82/88 [93%] AD vs 13/23 [56%] FTLD), and AD was frequently considered a copathology in FTLD-related syndromes (30/198 [15%]). Plasma p-tau217 concentrations were higher in AD-related syndromes (mean [SD], 0.28 [0.16] pg/mL) than FTLD-related syndromes (mean [SD], 0.10 [0.09] pg/mL) (*P* < .05). Plasma p-tau217 concentrations were highest in atypical AD-related syndromes (mean [SD], 0.33 [0.02] pg/mL), followed by typical late-onset amnestic syndromes (mean [SD], 0.27 [0.03] pg/mL). FTLD-related syndromes with AD (mean [SD], 0.19 [0.02] pg/mL) were higher compared to without (mean [SD], 0.07 [0.00] pg/mL). Plasma p-tau217 detected AD neuropathology across syndromes (area under the receiver operating characteristic curve [AUC], 0.95; 95% CI, 0.93-0.97), with slightly better performance in AD-related syndromes (AUC, 0.98; 95% CI, 0.95-1.00) compared to FTLD-related syndromes (AUC, 0.89; 95% CI, 0.83-0.94). NfL and GFAP had lower performance for detecting AD (AUC, 0.73; 95% CI, 0.68-0.78 and AUC, 0.75; 95% CI, 0.67-0.80, respectively) and added little to no diagnostic value either alone or in combinations with p-tau217. The presence of AD in FTLD-related syndromes was associated with lower Mini-Mental State Examination score (mean [SD], −2.90 [1.09]; *P* < .05), worse performance on memory (mean [SD] *z* score, −0.64 [0.32]), executive (mean [SD] *z* score, −0.74 [0.19]), and visuospatial composites (mean [SD] *z* score, −0.88 [0.37]) as well as increased rates of posterior cortical atrophy.

**Conclusion:**

Clinically relevant AD was prevalent across neurodegenerative syndromes and detectable with plasma p-tau217. Plasma p-tau217 may be a useful tool to investigate the clinical impact of AD copathology in non-AD neurodegenerative syndromes, including the effect of disease-modifying therapies.

## Key Points

**Question:**

What is the clinical value of blood-based biomarkers (plasma phosphorylated tau217 [p-tau217], glial fibrillary acidic protein [GFAP], and neurofilament light chain [NfL]) to detect Alzheimer disease (AD) in clinical syndromes related to frontotemporal lobar degeneration (FTLD)?

**Findings:**

In this cohort study including 349 individuals, AD neuropathology was common in FTLD-related syndromes and associated with worse clinical performance and differing patterns of atrophy. AD was accurately detected with plasma p-tau217, whereas NfL and GFAP added little value.

**Meaning:**

In this study, plasma p-tau217 detected clinically relevant AD in FTLD-related syndromes, which could support investigations into the impact of AD-targeted therapies in non-AD syndromes.

## Teaser

This cohort study evaluates the utility of 3 plasma biomarkers in detecting Alzheimer disease in clinical syndromes related to frontotemporal lobar degeneration.

## Introduction

The approval of disease-modifying treatments for Alzheimer disease (AD) have greatly increased the role of AD biomarkers in the clinic, and several blood-based biomarkers have emerged, often with extensive validation in detecting amyloid plaques and tau tangles in late-onset amnestic clinical syndromes, the primary intended context of use.^1,2,3,4,5,6,7^ In addition, as familiarity with blood-based biomarkers increases, clinical use is expected in neurodegenerative syndromes not typically associated with AD, such as frontotemporal dementia (FTD) spectrum syndromes, which are typically associated with underlying frontotemporal lobar degeneration (FTLD). The use of AD biomarkers in FTLD-related syndromes may be appropriate and even critical, as autopsy studies have shown that AD is frequently present as a copathology and may occasionally be the primary (driving) pathology.^8,9^ However, previous clinicopathological studies tying AD copathology to clinically relevant symptomatology in FTLD-related syndromes are limited,^8,9,10^ largely because investigations into these questions require autopsy due to the lack of confirmatory biomarkers of FTLD.

Three blood-based biomarkers increasingly used in clinical settings are tau phosphorylated at 217 (p-tau217), neurofilament light chain (NfL), and glial fibrillary acidic protein (GFAP).^11^ Plasma p-tau217 has emerged as a particularly promising biomarker for AD, demonstrating high specificity and sensitivity across studies, including those involving gold-standard autopsy confirmation.^1,2,3,4,5,6,7^ Similarly, plasma NfL concentrations have diagnostic value across a range of neurological disorders, serving as a nonspecific marker of neuroaxonal damage,^12^ whereas GFAP has been identified as a marker of astrocytic activation, reflecting neuroinflammatory processes linked to amyloid pathology.^13,14^ Recent research has also explored combinations of these biomarkers to enhance diagnostic accuracy,^15,16^ and these blood-based biomarkers have the potential to identify candidates for disease-modifying AD therapies. However, without consideration of the clinical context, a positive AD biomarker may lead to misattribution of all clinical symptoms to AD, even though in many syndromes an alternative neuropathology may be more clinically relevant. This misattribution might lead to misplaced confidence in how patients respond to treatment, especially as atypical presentations of AD and FTLD-related syndromes were not included in amyloid-targeting clinical trials.^17^ It remains an intriguing possibility that patients with non-AD neuropathology, who also have AD copathology, may benefit from disease-modifying AD therapies, but this is far from certain, especially as it is little known to what degree AD copathology contributes to clinical symptoms.

The University of California, San Francisco (UCSF) Neurodegenerative Disease Brain Bank is poised to address these questions due to a wide spectrum of neurodegenerative syndromes that have come to autopsy after antemortem clinical evaluation paired with blood banking, especially those with syndromes associated with FTLD. Better understanding the prevalence and role of AD in this cohort, and whether AD is detectable with blood biomarkers, would permit rapid identification of the AD in these syndromes, leading to new opportunities to parse contributions from various neuropathologies to clinical presentation.^18^ Therefore, we investigated whether plasma biomarkers could identify individuals with AD on autopsy in 12 clinical neurodegenerative syndromes associated with either AD or non-AD neuropathology to better understand the clinical value of testing for plasma p-tau217, GFAP, and NfL in these cohorts. The ability of these biomarkers to detect both amyloid and tau neuropathology and the added value of combining multiple blood biomarkers were evaluated.

## Methods

### Cohort Selection

This retrospective autopsy study included research participants evaluated between August 2008 and March 2021 at the UCSF Alzheimer Disease Research Center. Participants provided written informed consent at the time of enrollment, and studies were approved by the institutional review board at UCSF. This study followed the Strengthening the Reporting of Observational Studies in Epidemiology (STROBE) reporting guideline. All autopsied participants with clinical evaluation matched to blood collection were considered for inclusion (N = 370). Unclassifiable or nonneurodegenerative diagnoses (eg, psychiatric) were excluded (n = 21), resulting in a final cohort of 349: cognitively unimpaired (CU; n = 16), mild cognitive impairment (MCI; n = 19), late-onset amnestic AD (onset after age 65 years; LOAD; n = 35), early-onset amnestic AD (onset before age 65 years; EOAD; n = 36), logopenic variant primary progressive aphasia (lvPPA; n = 16), posterior cortical atrophy (PCA; n = 19), corticobasal syndrome (CBS; n = 35), progressive supranuclear palsy–Richardson syndrome (PSP-RS; n = 40), nonfluent variant PPA (nfvPPA; n = 21), semantic variant PPA (svPPA; n = 21), behavioral variant frontotemporal dementia (bvFTD; n = 76), amyotrophic lateral sclerosis (ALS; n = 5), and dementia with Lewy bodies (DLB; n = 10). For grouped analyses, AD-related syndromes (n = 125; MCI, LOAD, EOAD, lvPPA, and PCA) and FTLD-related syndromes (n = 198; CBS, PSP-RS, nfvPPA, svPPA, bvFTD, and ALS) were combined reflecting a priori assumptions of a hypothetical treating clinician.

### Clinical Assessment and Autopsy

All participants underwent a comprehensive clinical assessment. Clinical syndrome was diagnosed based on all available data at the time of clinical evaluation, following established diagnostic criteria,^19,20,21,22,23,24,25^ updated to follow contemporary nomenclature. Clinical disease severity was reported using the Mini-Mental State Examination (MMSE) and the Clinical Dementia Rating (CDR) plus the National Alzheimer Coordinating Center (NACC) FTLD (CDR+NACC FTLD).^26^ Domain-specific cognitive *z* scores were determined from a standardized neuropsychological battery (eMethods in Supplement 1).^27^ Post mortem brain tissue was analyzed between November 2008 and July 2022 by the UCSF Neurodegenerative Disease Brain Bank (NDBB). Neuropathological diagnosis sought to identify the primary neuropathological diagnosis (the one most closely linked to the clinical syndrome), the presence of contributing diagnoses (plausibly linked, anatomically, to a reported major symptom), and incidental diagnoses (without a clear link to a major symptom), in the opinion of the neuropathologist. Assessment of AD Neuropathological Change (ADNC), FTLD, and LBD followed standard protocols.^28,29,30^ AD was defined as present if intermediate or high ADNC was present.

### Neuroimaging

MRI acquisition and processing details are available in the eMethods in Supplement 1. Voxel-based time trajectories of gray matter and white matter atrophy were modeled using hierarchical empirical bayesian linear mixed-effects methods.^31^ Annualized atrophy rates were calculated and compared between groups after accounting for age and total intracranial volume.

### Plasma Biomarkers

Blood was collected in ethylenediaminetetraacetic acid (EDTA) tubes, centrifuged, and aliquoted into polypropylene tubes for −80 °C storage until sent for analyses (zero freeze/thaw cycles).^32^ Fasting state was not collected. Plasma p-tau217 was quantified with single-molecule array (SIMOA) technology as previously described.^27,33^ Plasma NfL and GFAP were quantified using commercially available SIMOA kits (Neurology 2-Plex B, Quanterix). Assays were performed by Quanterix in duplicate from the same aliquot in the same batch, blinded to sample identity. Coefficient of variation was less than 5% for each assay (eTable 2 in Supplement 1), which was unrelated to the age of sample. Analyses incorporated all data.

### Statistical Analysis

Clinical characteristics were compared across groups using linear or logistic regressions incorporating age, sex, and education as covariates for clinical severity comparisons or age, sex, and interval to autopsy (time between collection and autopsy) for biomarkers. Diagnostic performance was assessed using area under the receiver operating characteristic curve (AUC) using a binarized neuropathological variable as outcome and plasma biomarker(s) as predictor, covaried for age, sex, and interval to autopsy. Youden index cutoffs were determined for each biomarker, and diagnostic performance was assessed across clinical syndromes. For comparisons with multiple plasma biomarker predictors, AUCs were calculated for each model and compared via DeLong tests.^34^ Analyses were performed in R version 4.2.1 (R Foundation).

## Results

### Clinical Characteristics

Characteristics of 349 autopsied individuals (191 [55%] male and 158 [45%] female; mean [SD] age at death, 72 [11] years) are presented in the Table, including demographic characteristics, clinical severity, AD neuropathology, and plasma biomarker concentrations, displayed by clinical syndrome at time of evaluation and combined into cohorts representing AD-related (n = 125) and FTLD-related clinical syndromes (n = 198). Of note, CU individuals (n = 16), recruited through healthy aging studies, were older on average at time of assessment and death than neurodegenerative cohorts, and half had possibly clinically relevant neuropathology at autopsy that may have been present at evaluation or developed in the mean (SD) 2.9 (2.8)–year interval. Individuals with AD-related syndromes were older at time of assessment and death than those with FTLD-related syndromes, and individuals with FTLD-related syndromes had a shorter interval to autopsy than those with AD-related syndromes (mean [SD], 3.8 [2.2] vs 3.1 [2.3] years; *P* < .05), consistent with known earlier onset and faster disease course in FTLD. Sex, education, and race and ethnicity did not differ between cohorts. MMSE scores were lower in individuals with AD-related syndromes than in those with FTLD-related syndromes, but disease severity, measured by CDR+NACC FTLD global and box scores, did not differ, although neurodegenerative cohorts had higher CDR+NACC FTLD than CU individuals.

**Table.** Cohort Characteristics^a^

Clinical syndrome	No. (%)														
	All		CU	AD-related syndromes					FTLD-related syndromes						DLB
	AD-related syndromes	FTLD-related syndromes		MCI	LOAD	EOAD	lvPPA	PCA	CBS	PSP-RS	nfvPPA	svPPA	bvFTD	ALS	
No.	125	198	16	19	35	36	16	19	35	40	21	21	76	5	10
Demographic characteristics															
Age at evaluation, mean (SD),y	70 (11)^b^	67 (9)^b^	86 (6)	81 (9)	78 (6)^c^	61 (7)^c^	65 (10)^c^	66 (8)^c^	70 (8)^c^	70 (7)^c^	72 (6)^c^	68 (6)^c^	62 (10)^c^	63 (9)^c^	71 (6)^c^
Age at death, mean (SD), y	73 (11)^b^	69 (10)^b^	89 (7)	82 (10)	82 (6)^c^	65 (7)^c^	67 (10)^c^	70 (8)^c^	72 (8)^c^	72 (7)^c^	75 (6)^c^	74 (7)^c^	64 (11)^c^	64 (10)^c^	74 (7)^c^
Sex															
Male	71 (57)	107 (54)	7 (44)	14 (74)	20 (57)	19 (53)	5 (31)	13 (68)	18 (51)	20 (50)	10 (48)	10 (48)	46 (61)	3 (60)	6 (60)
Female	54 (43)	91 (46)	9 (56)	5 (26)	15 (43)	17 (47)	11 (69)	6 (32)	17 (49)	20 (50)	11 (52)	11 (52)	30 (39)	2 (40)	4 (40)
Education, mean (SD), y	17 (3)	17 (7)	18 (2)	17 (4)	17 (3)	16 (3)	17 (4)	17 (3)	20 (15)	16 (3)	17 (3)	17 (3)	16 (3)	17 (2)	16 (2)
Race and ethnicity^d^															
American Indian	0	2 (1)	0	0	0	0	0	0	0	0	0	1 (5)	1 (1)	0	0
Asian	2 (2)	4 (2)	0	0	0	1 (3)	1 (6)	0	1 (3)	1 (2)	1 (5)	0	1 (1)	0	0
Black	2 (2)	1 (1)	0	0	0	1 (3)	1 (6)	0	1 (3)	0	0	0	0	0	0
Hispanic	6 (5)	7 (4)	1 (6)	2 (11)	0	3 (8)	1 (6)	0	0	4 (10)	1 (5)	0	2 (3)	0	1 (10)
Non-Hispanic White	111 (89)	175 (88)	15 (94)	17 (89)	35 (100)	28 (78)	12 (75)	19 (100)	29 (83)	34 (85)	19 (90)	18 (86)	70 (92)	5 (100)	7 (70)
Not reported	4 (3)	9 (5)	0	0	0	3 (8)	1 (6)	0	4 (11)	1 (2)	0	2 (10)	2 (3)	0	2 (20)
Clinical severity															
MMSE score, mean (SD)	18 (7)^b^	21 (7)^b^	29 (1)	26 (5)	19 (5)^c^	14 (7)^c^	13 (8)^c^	18 (6)^c^	21 (8)^c^	25 (5)	22 (8)^c^	20 (7)^c^	19 (8)^c^	27 (2)	23 (6)
CDR+NACC FTLD global score, mean (SD)	1.8 (0.9)	1.9 (0.9)	0.1 (0.2)	0.7 (0.4)^c^	2.2 (0.8)^c^	2.0 (0.8)^c^	1.5 (0.7)^c^	1.8 (0.8)^c^	1.6 (0.9)^c^	1.7 (0.9)^c^	1.8 (0.9)^c^	2.0 (0.8)^c^	2.2 (0.7)^c^	1.1 (0.9)^c^	1.4 (0.7)^c^
CDR+NACC FTLD box score, mean (SD)	10.9 (6.7)	11.9 (6.4)	0.1 (0.2)	3.1 (2.6)	13.5 (6.1)^c^	12.8 (7.1)^c^	8.9 (4.9)^c^	11.5 (5.3)^c^	9.2 (6.2)^c^	8.9 (5.6)^c^	10.5 (6.8)^c^	12.8 (6.5)^c^	15.1 (5.1)^c^	5.5 (5.0)	8.5 (4.9)^c^
Neuropathology															
AD present	110 (88)	45 (23)	6 (38)	9 (47)	32 (91)	35 (97)	15 (94)	19 (100)	14 (40)	11 (28)	6 (29)	4 (19)	10 (13)	0	7 (70)
ADNC score, mean (SD)	2.7 (0.8)^b^	1.1 (0.9)^b^	1.3 (1.1)	1.5 (1.0)	2.8 (0.6)^c^	2.9 (0.3)^c^	2.8 (0.8)^c^	2.9 (0.2)^c^	1.5 (1.1)	1.1 (0.9)	1.2 (1.0)	1.2 (1.0)	0.8 (0.8)^c^	0.6 (0.5)	2.3 (0.9)^c^
Not	4 (3)	54 (27)	4 (25)	3 (16)	0	0	1 (6)	0	7 (20)	9 (23)	5 (24)	4 (19)	27 (36)	2 (40)	0
Low	11 (9)	99 (50)	6 (38)	7 (37)	3 (9)	1 (3)	0	0	14 (40)	20 (5)	10 (48)	13 (62)	29 (51)	3 (60)	3 (30)
Intermediate	8 (6)	20 (10)	3 (19)	5 (26)	1 (3)	0	1 (6)	1 (5)	4 (11)	8 (20)	3 (14)	0	5 (7)	0	1 (10)
High	102 (82)	25 (13)	3 (19)	4 (21)	31 (89)	35 (97)	14 (88)	18 (95)	10 (29)	3 (8)	3 (14)	4 (19)	5 (7)	0	6 (60)
Thal phase, mean (SD)	4.4 (1.3)^b^	1.7 (1.7)^b^	2.2 (1.8)	2.4 (1.7)	4.6 (1.0)^c^	4.9 (0.4)^c^	4.7 (1.2)^c^	4.9 (0.3)^c^	2.4 (2.0)	1.9 (1.6)	2.1 (1.7)	1.8 (1.6)	1.3 (1.5)^c^	0.6 (0.5)	4.1 (1.4)^c^
Braak stage, mean (SD)	5.4 (1.3)^b^	2.3 (1.7)^b^	3.3 (1.2)	3.7 (1.7)	5.5 (1.4)^c^	5.8 (0.9)^c^	5.8 (0.5)^c^	5.8 (0.7)^c^	3.2 (2.2)	2.2 (1.3)^c^	2.8 (1.7)	2.1 (1.8)^c^	1.8 (1.6)^c^	1.2 (0.8)^c^	4.2 (1.9)
Plasma biomarkers															
P-tau217, mean (SD), pg/mL	0.28 (0.16)^b^	0.10 (0.09)^b^	0.10 (0.08)	0.12 (0.07)	0.27 (0.16)^c^	0.33 (0.15)^c^	0.33 (0.17)^c^	0.32 (0.15)^c^	0.13 (0.11)	0.10 (0.06)	0.11 (0.12)	0.09 (0.12)	0.08 (0.06)	0.08 (0.04)	0.12 (0.07)
NfL, mean (SD), pg/mL	31 (28)^b^	50 (50)^b^	32 (14)	32 (19)	34 (19)	26 (30)	39 (52)	29 (13)	63 (93)^c^	36 (20)	38 (16)	41 (20)^c^	57 (45)^c^	61 (20)	17 (4)
GFAP, mean (SD), pg/mL	267 (131)^b^	201 (155)^b^	235 (67)	204 (90)	292 (123)^c^	259 (131)^c^	285 (197)^c^	286 (94)^c^	235 (177)	203 (144)	209 (100)	190 (127)	189 (172)	128 (63)	214 (56)

Abbreviations: AD, Alzheimer disease; ADNC, AD neuropathological change; ALS, amyotrophic lateral sclerosis; bvFTD, behavioral variant frontotemporal dementia; CBS, corticobasal syndrome; CDR, Clinical Dementia Rating; CU, cognitively unimpaired; DLB, dementia with Lewy bodies; EOAD, early-onset Alzheimer disease; FTLD, frontotemporal lobar degeneration; GFAP, glial fibrillary acidic protein; LOAD, late-onset Alzheimer disease; lvPPA, logopenic variant primary progressive aphasia; MCI, mild cognitive impairment; MMSE, Mini-Mental State Examination; NACC, National Alzheimer’s Coordinating Center; NfL, neurofilament light chain; nfvPPA, nonfluent variant primary progressive aphasia; PCA, posterior cortical atrophy; PSP-RS, progressive supranuclear palsy–Richardson syndrome; p-tau217, phosphorylated tau 217; svPPA, semantic variant primary progressive aphasia.

^a^ Statistical comparisons used regression with age, sex, and education as covariates for clinical severity comparisons and age, sex, and interval to autopsy as covariates for biomarker comparisons, comparing between AD-related and FTLD-related syndromes (*P* < .05) or comparing each clinical symptom to CU (*P* < .05). For calculation of means, ADNC was coded as 0 = not, 1 = low, 2 = intermediate, 3 = high.

^b^ *P* < .05.

^c^ *P* < .05.

^d^ Race and ethnicity data were self-reported by study participants. At the University of California, San Francisco, race, ethnic group, and ethnicity data are collected from patients using the We Ask Because We Care form. Reporting race and ethnicity in this study was mandated by the National Institutes of Health, consistent with the inclusion of women, minorities, and children policy. Race and ethnicity were collected to assess equity in participation and generalizability of results.

### Neuropathological Distribution of AD Primary and Copathology Across Neurodegenerative Clinical Syndromes

**Figure 1.**
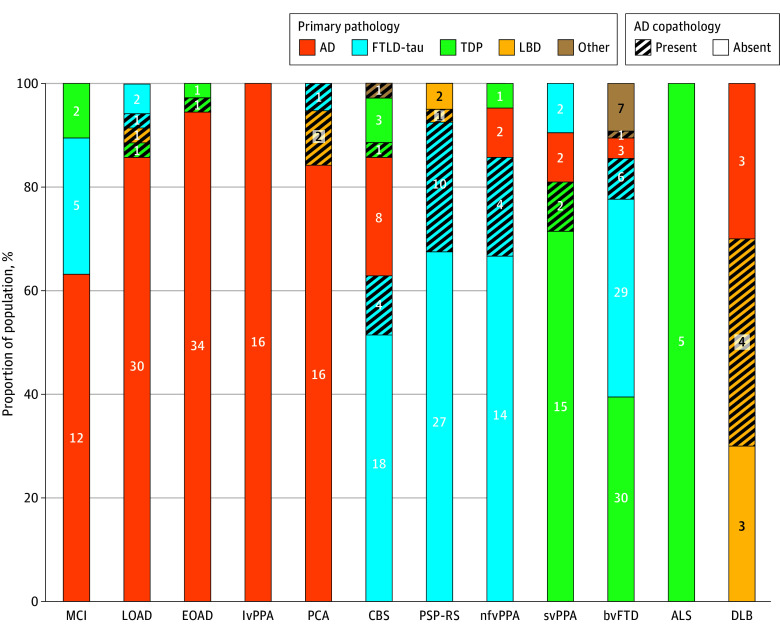
Neuropathological Distribution of Alzheimer Disease (AD) Primary and Copathology Across Neurodegenerative Clinical Syndromes Primary neuropathology was defined by an expert neuropathologist. AD copathology was defined as present if intermediate or high AD neuropathological changes were present. For corticobasal syndrome (CBS), other pathology included 1 case of Huntington disease; for behavioral variant frontotemporal dementia (bvFTD), other pathology comprised 8 cases of rarer pathologies, including frontotemporal lobar degeneration (FTLD) fused in sarcoma (n = 4), FTLD with ubiquitinated inclusions (n = 2), FTLD with no inclusions (n = 1), and cerebral amyloid angiopathy (n = 1). Numbers in bars indicate case counts. ALS indicates amyotrophic lateral sclerosis; DLB, dementia with Lewy bodies; EOAD, early-onset Alzheimer disease; LBD, Lewy body dementia; LOAD, late-onset Alzheimer disease; lvPPA, logopenic variant primary progressive aphasia; MCI, mild cognitive impairment; nfvPPA, nonfluent variant primary progressive aphasia; PCA, posterior cortical atrophy; PSP-RS, progressive supranuclear palsy–Richardson syndrome; svPPA, semantic variant primary progressive aphasia; TDP, transactive response DNA-binding protein.

AD was present in 167 of 349 individuals (67%), including 110 of 125 with AD-related syndromes (88%), 45 of 198 with FTLD-related syndromes (23%), and 7 of 10 with DLB (70%). Excluding MCI, 92 of 101 AD-related syndromes (91%) had underlying AD as the primary neuropathological diagnosis (Figure 1). The prevalence of AD as the primary pathology was highest in lvPPA (16/16 [100%]), followed by EOAD (34/36 [94.4%]), LOAD (30/35 [85.7%]), and PCA (16/19 [84.2%]). All AD-related syndromes where the primary pathology was not AD did have AD present as a copathology, except for 2 LOAD cases (Pick disease and chronic traumatic encephalopathy) and 1 EOAD case with FTLD-TDP-A related to a pathogenic variant in the *GRN* gene. In FTLD-related syndromes, the prevalence of AD neuropathology was variable across clinical syndromes: CBS (14/35 [40%]), nfvPPA (6/21 [29%]), PSP-RS (11/40 [28%]), svPPA (4/21 [19%]), bvFTD (10/76 [13%]), with no AD copathology observed in ALS (0/5), and the percentage of cases where AD was considered primary was lower: CBS (8/35 [23%]), nfvPPA (2/21 [10%]), svPPA (2/21 [10%]), bvFTD (3/76 [5%]), PSP-RS (0/40), and ALS (0/5). In FTLD-related syndromes, when AD was present, it was frequently intermediate ADNC (20/45 [44%]) and more likely to be considered copathology (30/45 [67%]). Full neuropathological characteristics for the cohort are available in eTable 1 in Supplement 1.

### Plasma Biomarker Concentrations Across Clinical Syndromes

**Figure 2.**
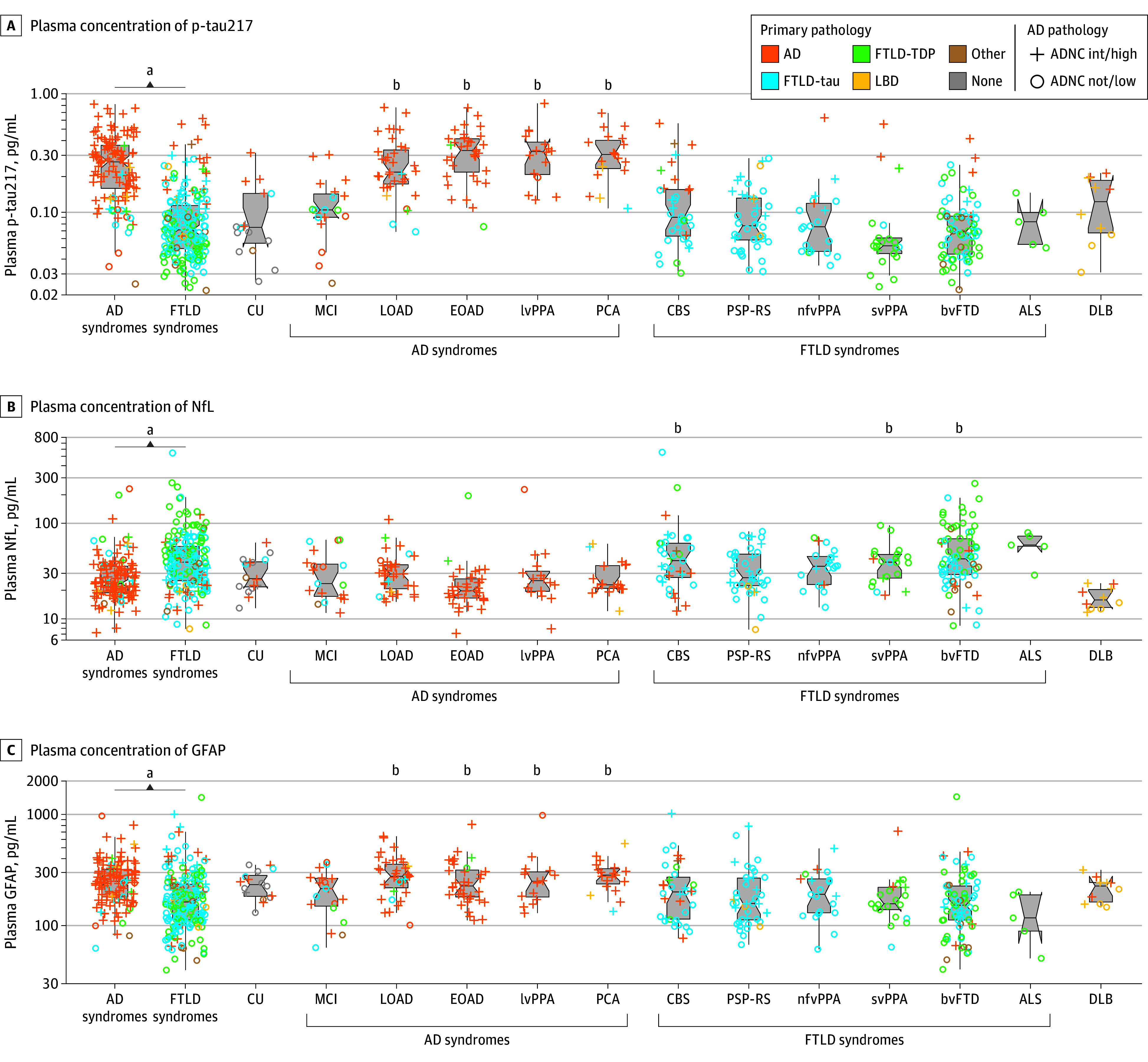
Plasma Biomarker Concentrations Across Neurodegenerative Clinical Syndromes Plasma concentrations of (A) plasma phosphorylated tau217 (p-tau217), (B) neurofilament light chain (NfL), and (C) glial fibrillary acidic protein (GFAP) for each clinical neurodegenerative syndrome and grouped into Alzheimer disease (AD)– and frontotemporal lobar degeneration (FTLD)–related syndromes. Statistical comparisons used linear regression (age, sex, and interval to autopsy as covariates). ADNC indicates AD neuropathological change; ALS, amyotrophic lateral sclerosis; bvFTD, behavioral variant frontotemporal dementia; CBS, corticobasal syndrome; CU, cognitively unimpaired; DLB, dementia with Lewy bodies; EOAD, early-onset Alzheimer disease; LBD, Lewy body dementia; LOAD, late-onset Alzheimer disease; lvPPA, logopenic variant primary progressive aphasia; MCI, mild cognitive impairment; nfvPPA, nonfluent variant primary progressive aphasia; PCA, posterior cortical atrophy; PSP-RS, progressive supranuclear palsy–Richardson syndrome; svPPA, semantic variant primary progressive aphasia; TDP, transactive response DNA-binding protein. ^a^Different from each other (*P* < .05). ^b^Different from CU (*P* < .05).

**Figure 3.**
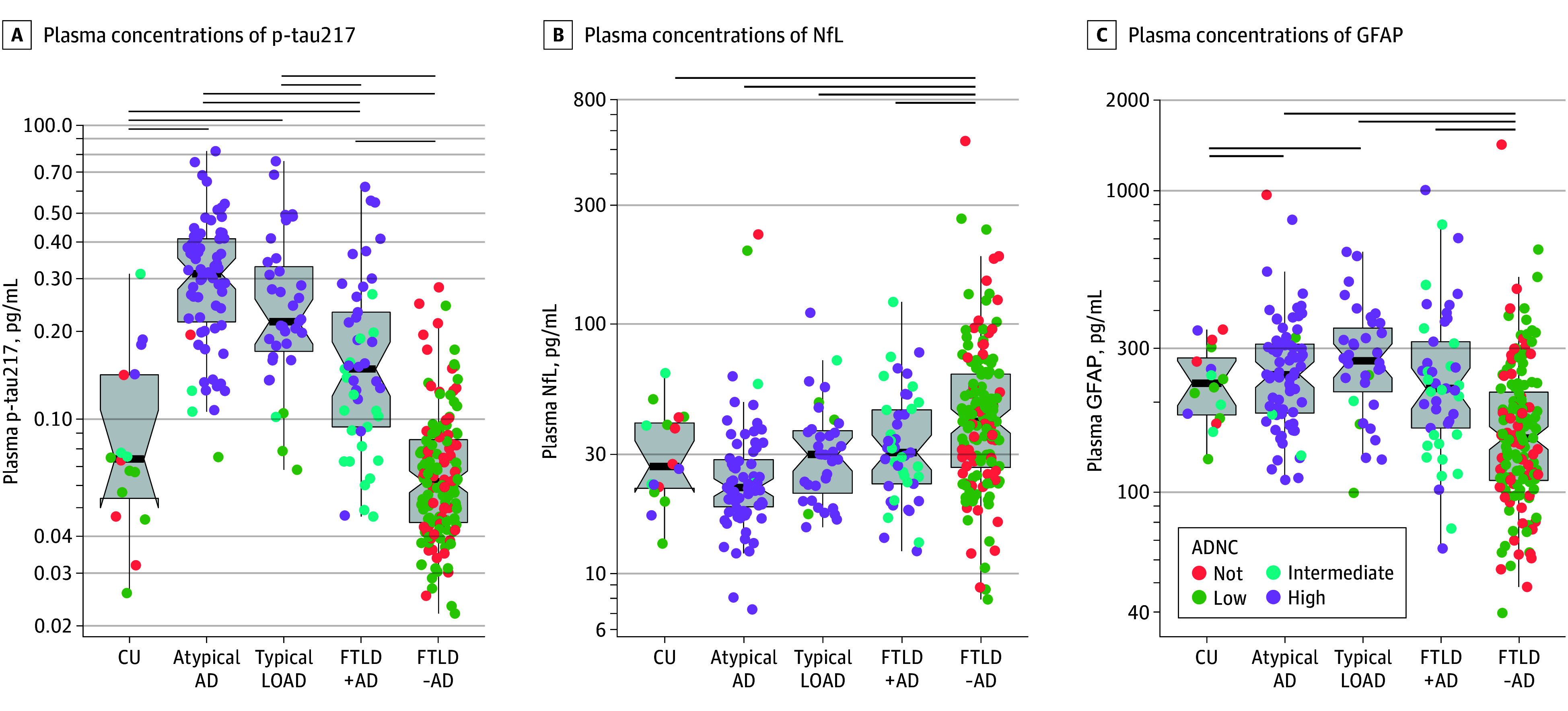
Plasma Biomarker Concentrations Comparing Atypical Alzheimer Disease (AD), Typical Late-Onset AD, and Frontotemporal Lobar Degeneration (FTLD)–Related Syndromes With and Without AD Neuropathology Plasma concentrations of (A) plasma phosphorylated tau217 (p-tau217), (B) neurofilament light chain (NfL), and (C) glial fibrillary acidic protein (GFAP) for each cohort. Statistical comparisons used linear regression (age, sex, and interval to autopsy as covariates); lines represent significant differences (*P* < .05). Color represents AD neuropathological change. ADNC indicates AD neuropathological change; CU, cognitively unimpaired; LOAD, late-onset Alzheimer disease.

Plasma p-tau217 was higher in AD-related syndromes (mean [SD], 0.28 [0.16] pg/mL) than in FTLD-related syndromes (mean [SD], 0.10 [0.09] pg/mL; *P* < .05) (Figure 2A; Table). Within AD-related syndromes, atypical AD syndromes (EOAD, lvPPA, and PCA) had the highest p-tau217 concentrations (mean [SD], 0.33 [0.2] pgm/L), followed by typical LOAD (mean [SD], 0.27 [0.03] pg/mL) (Figure 3A), whereas FTLD-related syndromes with AD had higher p-tau217 concentrations than FTLD-related syndromes without AD (mean [SD], 0.19 [0.02] pg/mL vs mean [SD], 0.07 [0.00] pg/mL) (Figure 3A). Regression analysis measured effect size of increasing age (β, −0.03; 95% CI, −0.13 to 0.12; *P* = .61), female sex (β, 0.10; 95% CI, 0.03 to 0.19; *P* < .05), and longer autopsy intervals (β, 0.03; 95% CI, −0.06 to 0.12; *P* = .48), and whereas female sex had a small effect size on p-tau217 (higher concentrations), sensitivity analyses removing sex did not change overall results. Within clinical syndromes, p-tau217 was higher in those with AD neuropathology vs no AD neuropathology in both FTLD-related syndromes and AD-related syndromes (eFigure 1A in Supplement 1).

As expected, NfL was higher in FTLD-related syndromes (mean [SD], 50 [50] pg /mL) compared to AD-related syndromes **(**mean [SD], 31 [28] pg /mL) (Figures 2B and 3B; Table), whereas GFAP was higher in AD-related syndromes (mean [SD], 267 [131] pg /mL vs mean [SD], 201 [155] pg /mL) (Figures 2C and 3C; Table). For NfL, female sex (β, 0.16; 95% CI, 0.06 to 0.26; *P* < .05) and longer interval to autopsy (β, −0.24; 95% CI, −0.35 to −0.14; *P* < .05) had small effect sizes, but not older age (β, 0.05; 95% CI, −0.08 to 0.18; *P* = .44); whereas for GFAP, both older age (β, 0.21; 95% CI, 0.07 to 0.34; *P* < .05) and female sex (β, 0.20; 95% CI, 0.10 to 0.30; *P* < .05) had small effect sizes, but not a longer autopsy interval (β, −0.04; 95% CI, −0.15 to 0.07; *P* = .45). NfL was also higher in those with AD neuropathology vs no AD neuropathology within AD-related clinical syndromes, but not within FTLD-related syndromes (eFigure 1B in Supplement 1). GFAP was also higher in FTLD-related syndromes with AD vs without (eFigure 1C in Supplement 1).

### Diagnostic Performance of Plasma Biomarkers to Detect AD Neuropathology

Plasma p-tau217 had excellent diagnostic accuracy in predicting intermediate or higher ADNC across the entire cohort (AUC, 0.95; 95% CI, 0.93-0.97), with better diagnostic performance in AD-related syndromes (AUC, 0.98; 95% CI, 0.95-1.00) compared to FTLD-related syndromes (AUC, 0.89; 95% CI, 0.83-0.94) (eFigure 2 in Supplement 1). Across the entire cohort, plasma p-tau217 was accurate in detecting AD neuropathology (ie, ADNC), including both amyloid (ie, Thal phase and Consortium to Establish a Registry of Alzheimer Disease [CERAD] score) and tau (ie, Braak stages), with the best diagnostic performance being detection of high ADNC, Thal phase greater than 3, Braak stage greater than 4, and frequent CERAD neuritic plaques (eFigures 2 and 3 in Supplement 1). Youden index identified an optimum plasma p-tau217 cutoff for detecting AD across the entire cohort: 0.125 pg/mL. Diagnostic performance to detect AD (primary or copathology) was calculated for each biomarker in each clinical syndrome (eFigure 4 and eTable 3 in Supplement 1), with diagnostic performance generally lower for FTLD-related syndromes (although this should be cautiously interpreted, given low prevalence in certain syndromes).

In contrast, NfL and GFAP had worse diagnostic performance compared to p-tau217 in the entire cohort (NfL AUC, 0.73; 95% CI, 0.68-0.78; GFAP AUC, 0.75; 95% CI, 0.67-0.80), with slightly better performance in AD-related syndromes than in FTLD-related syndromes (eFigure 2 in Supplement 1). Detection was equally poor for both amyloid and tau, but better detection of high ADNC and Braak stages was observed within AD-related syndromes where lower NfL values were predictive of AD as higher NfL values were identifying FTLD mimics. Generally, NfL was elevated in lower stages of AD neuropathology, reflecting the higher likelihood of FTLD in this cohort (eFigure 5 in Supplement 1). GFAP concentrations were elevated in higher levels of amyloid neuropathology (CERAD frequent, Thal phase 4 or higher), but the fold-change over higher ADNC and Braak stages was modest compared to p-tau217 (eFigure 6 in Supplement 1).

### Plasma Biomarker Combinations to Detect AD Neuropathology

To investigate the value of combining plasma biomarkers to detect AD neuropathology, we analyzed diagnostic performance across the entire cohort for each plasma biomarker alone and in combination (eFigure 7 in Supplement 1). All analyses included age, sex, and interval to autopsy as covariates; sensitivity analyses performed excluding these covariates did not differ. As above, plasma p-tau217 alone (AUC, 0.95) demonstrated significantly higher AUC values compared to the plasma NfL (AUC, 0.73) or GFAP (AUC, 0.75) alone, or NfL+GFAP in combination (AUC, 0.86) when p-tau217 was not included. Across all combinations, the highest numerical AUC values were observed with the combination of p-tau217+NfL (AUC, 0.96), which did not differ from p-tau217+NfL+GFAP (AUC, 0.96), but these combinations were not statistically different from plasma p-tau217 alone (eTable 4 in Supplement 1).

### Clinical Relevance of AD Neuropathology in FTLD-Related Clinical Syndromes

**Figure 4.**
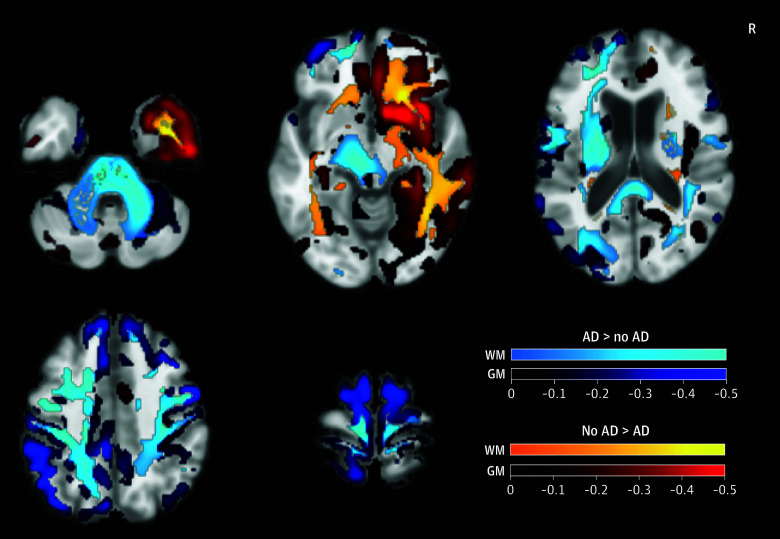
Alzheimer Disease (AD) Copathology in Frontotemporal Lobar Degeneration (FTLD)–Related Syndromes and Increased Rates of Posterior Cortical Atrophy Voxel-based bayesian linear mixed effects showing differences in rates of atrophy on magnetic resonance imaging in white matter (WM) and gray matter (GM) regions over time in FTLD syndromes with or without AD. Statistical analyses controlled for age and total intracranial volume, and results are masked to show only statistically significant regions (*P* < .05) after familywise error correction.

As the clinical importance of AD in FTLD-related clinical syndromes is poorly understood, linear regressions were used to determine the effect of AD on neuropsychological testing, controlling for age, sex, and education (eTable 5 in Supplement 1). Within FTLD-related syndromes, the presence of AD was associated with lower MMSE (mean [SD], −2.90 [1.09]) and worse performance on memory (mean [SD] *z* score, −0.64 [0.32]), executive (mean [SD] *z* score, −0.74 [0.19]), and visuospatial domains (mean [SD] *z* score, −0.88 [0.37]) but not language (mean [SD] *z* score, −0.26 [0.46]). Finally, rates of atrophy on MRI were compared within FTLD-related clinical syndromes, stratified by the presence or absence of AD. FTLD cases with AD copathology showed higher rates of posterior atrophy, whereas pure FTLD syndromes showed higher rates of atrophy in regions typical for FTLD, such as the right anterior temporal lobe (Figure 4).

## Discussion

In this cohort study, AD was common and most accurately detected by p-tau217 alone across a diverse spectrum of neurodegenerative syndromes, not only in clinical syndromes commonly associated with AD, but also in clinical syndromes typically associated with FTLD neuropathology. The relatively high prevalence of clinically relevant AD copathology highlights that relying solely on AD biomarkers to determine the likely cause of dementia may result in misdiagnosis or misattribution errors if clinical features are not part of diagnostic algorithms, especially in neurodegenerative syndromes where other neuropathologies are more frequently causative. However, the relatively accurate detection of AD across clinical syndromes provides an opportunity to understand the clinical relevance of AD neuropathology in these patients, including whether they would potentially benefit from disease-modifying therapeutics that have evidence of efficacy in typical amnestic AD presentations. Therefore, our findings support the use of AD biomarker testing for any patient presenting with cognitive and behavioral concerns, but clinical features must be factored into interpretation of results and treatment decisions. Intriguingly, female sex also had a small positive effect size on all biomarkers, in line with our previous findings in CU cohorts.^27^

Similar to previous studies,^1,2,3,4,5,6,7^ including our own, we found excellent diagnostic detection of primary AD neuropathology using p-tau217, even when the clinical phenotype was not AD related. However, the diagnostic accuracy of p-tau217 did vary slightly by clinical syndrome, likely driven by the proportion of cases with intermediate ADNC (often copathology), where p-tau217 concentrations are less elevated and diagnostic performance is lower, highlighting that p-tau217 may be best used in symptomatic patients with higher or detectable levels of neuropathology. Intriguingly, not only was AD detectable by p-tau217 in FTLD-related syndromes, but the presence of AD was associated with worse cognitive performance and different patterns of atrophy, suggesting clinically relevance. Herein, we also replicated prior studies of the differential diagnostic value of plasma p-tau217 for AD, including our own work using the Lilly assay on the MSD platform,^1^ with a clinically available p-tau217 assay on the SIMOA platform.^1,33^ The p-tau217 immunoassay used in this study has shown comparable performance to other immunoassays in head-to-head studies detecting amyloid measured by positron emission tomography.^35^ Future studies with simultaneous biomarker measurement of all neuropathologies (eg, Lewy bodies and transactive response DNA-binding protein 43) would further clarify the relative contribution of each neuropathology to clinical features, although this is not currently possible outside of autopsy studies due to a lack of validated biomarkers for non-AD neuropathologies.

This large autopsy cohort with diverse neuropathologic etiologies permitted evaluation of the relative diagnostic performance to detect AD across the spectrum of AD stages, including cases with little to no AD neuropathology. Similar to numerous prior studies,^3,4,5,6^ this allowed us to demonstrate that plasma p-tau217 accurately detected both amyloid plaques and tau neurofibrillary tangles, even when other neuropathology was present. We also found increasing accuracy at higher levels of neuropathology, with the best detection occurring at neuropathological stages associated with symptomatic disease. These findings support the clinical use of plasma p-tau217 in patients with cognitive or behavioral symptoms to diagnose clinically relevant AD, including both amyloid and tau.

Similar to previous studies,^36,37^ plasma NfL showed comparatively lower diagnostic performance to detect AD in the overall cohort and in diagnostic clinical subgroups. However, NfL had lower concentrations in AD-related syndromes compared to FTLD-related syndromes, which may have diagnostic value, and we cannot exclude that NfL may have diagnostic utility, perhaps in combination with p-tau217 when FTLD is also on the differential, as in corticobasal syndrome. GFAP has been associated with amyloid neuropathology and suggested to have a role in early AD detection^38,39^; we found GFAP concentrations were higher in AD-related syndromes, but overall had poor diagnostic performance due to a comparatively small percentage increase. However, we acknowledge that this study cannot fully examine this context of use, as it lacks large numbers of asymptomatic and mild stages along the AD continuum (without other copathologies).

### Limitations

An important limitation of this study is shared with all clinicopathological comparisons, namely that individuals’ antemortem clinical features and plasma concentrations were compared to autopsy findings with variable intervals, sometimes spanning many years, between assessments. Our analyses controlled for interval to autopsy, but it is not possible to know whether the neuropathology on which autopsy diagnoses are based was present at the time of assessment or developed afterwards. Nonetheless, neuropathology remains the gold standard for biomarker validation, as it avoids errors introduced when biomarkers are validated against other, sometimes imperfect, biomarkers, especially when all neuropathologies present are relevant. An additional limitation is that the cohort was drawn from observational research, which introduces referral bias, especially as our center specializes in atypical AD- and FTLD-associated neurodegeneration. A further limitation is the lack of racial and ethnic diversity in this cohort, and although we expect the basic biology to be shared across groups, conflicting results on the impact of race on plasma biomarkers suggests we must replicate these results in a real-world clinical setting with clinic-grade assays in a diverse population.^40,41,42^

## Conclusions

In conclusion, AD neuropathology was common across many neurodegenerative presentations, both as primary and copathology. Plasma p-tau217 demonstrated high diagnostic accuracy to identify the presence of clinically relevant AD across a diverse spectrum of symptomatic patients, encompassing not only AD-related syndromes but also FTLD-related syndromes. In contrast, GFAP and NfL showed comparatively little diagnostic value to detect AD in this symptomatic cohort. These findings suggest that plasma p-tau217 may be a useful clinical tool for detection of AD neuropathology even in neurodegenerative syndromes not commonly associated with AD, potentially enabling evaluation of the effects of disease-modifying treatments as interventions to reduce AD copathology in the setting of other neurodegenerative diseases.

## Notes

Supplement 1. eMethods eTable 1. Neuropathological distribution of primary pathology and AD co-pathology in each clinical syndrome eTable 2. Technical performance in plasma biomarker assays eFigure 1. Plasma Biomarkers Across Clinical Neurodegenerative Syndromes by Presence/Absence of AD Neuropathology eFigure 2. Diagnostic Performance of Plasma Biomarkers to Detect AD-related Neuropathology eFigure 3. Plasma concentrations of p-tau217 across neuropathological stages eFigure 4. Diagnostic performance of plasma biomarkers for AD neuropathology across clinical neurodegenerative syndromes eTable 3. Diagnostic performance of plasma biomarkers for AD neuropathology across clinical diagnosis eFigure 5. Plasma concentrations of NfL across neuropathological stages eFigure 6. Plasma concentrations of GFAP across neuropathological stages eFigure 7. Diagnostic Value of Plasma Biomarker Combinations to Detect AD Neuropathology eTable 4. DeLong Comparison Across Plasma Biomarker Combinations eTable 5. Comparison of Neuropsychological Testing in FTLD-related syndromes with and without AD neuropathology

Supplement 2. Data sharing statement
