# Supplementary material for: Detection of Alzheimer Neuropathology in Alzheimer and Non-Alzheimer Clinical Syndromes With Blood-Based Biomarkers
Source: JAMA Neurol. 2025 Feb 10;82(4):344–54. doi: 10.1001/jamaneurol.2024.5017 (PMC11811866; doi:10.1001/jamaneurol.2024.5017)
Supplement: Supplement 1. — eMethods eTable 1. Neuropathological distribution of primary pathology and AD co-pathology in each clinical syndrome eTable 2. Technical performance in plasma biomarker assays eFigure 1. Plasma Biomarkers Across Clinical Neurodegenerative Syndromes by Presence/Absence of AD Neuropathology eFigure 2. Diagnostic Performance of Plasma Biomarkers to Detect AD-related Neuropathology eFigure 3. Plasma concentrations of p-tau217 across neuropathological stages eFigure 4. Diagnostic performance of plasma biomarkers for AD neuropathology across clinical neurodegenerative syndromes eTable 3. Diagnostic performance of plasma biomarkers for AD neuropathology across clinical diagnosis eFigure 5. Plasma concentrations of NfL across neuropathological stages eFigure 6. Plasma concentrations of GFAP across neuropathological stages eFigure 7. Diagnostic Value of Plasma Biomarker Combinations to Detect AD Neuropathology eTable 4. DeLong Comparison Across Plasma Biomarker Combinations eTable 5. Comparison of Neuropsychological Testing in FTLD-related syndromes with and without AD neuropathology [file jamaneurol-e245017-s001.pdf]

Supplemental Online Content

VandeVrede L, Cho H, Sanderson-Cimino M, et al. Detection of Alzheimer neuropathology in Alzheimer and non-Alzheimer clinical syndromes with blood-based biomarkers. *JAMA Neurol*. Published online February 10, 2025. doi:10.1001/jamaneurol.2024.5017

eMethods

eTable 1. Neuropathological distribution of primary pathology and AD co-pathology in each clinical syndrome

eTable 2. Technical performance in plasma biomarker assays

eFigure 1. Plasma Biomarkers Across Clinical Neurodegenerative Syndromes by Presence/Absence of AD Neuropathology

eFigure 2. Diagnostic Performance of Plasma Biomarkers to Detect AD-related Neuropathology

eFigure 3. Plasma concentrations of p-tau217 across neuropathological stages

eFigure 4. Diagnostic performance of plasma biomarkers for AD neuropathology across clinical neurodegenerative syndromes

eTable 3. Diagnostic performance of plasma biomarkers for AD neuropathology across clinical diagnosis

eFigure 5. Plasma concentrations of NfL across neuropathological stages

eFigure 6. Plasma concentrations of GFAP across neuropathological stages

eFigure 7. Diagnostic Value of Plasma Biomarker Combinations to Detect AD Neuropathology

eTable 4. DeLong Comparison Across Plasma Biomarker Combinations

eTable 5. Comparison of Neuropsychological Testing in FTLD-related syndromes with and without AD neuropathology

This supplemental material has been provided by the authors to give readers additional information about their work.

© 2025 VandeVrede L et al. *JAMA Neurology*.

eMethods

Neuropsychological Assessment

Cognitive testing protocols were harmonized across cohorts, with the exception of verbal episodic memory. Raw test scores were converted to domain-specific z scores based on the score distribution of a healthy aging cohort at UCSF (all CDR = 0; n > 650 per test). Cognition in each cohort was operationalized with composite z scores in four separate domains. Verbal episodic memory in the CU cohort was quantified via a composite of primary metrics from the California Verbal Learning Test, second edition (CVLT-II: total immediate recall, total long [20 minute] delay free recall, and recognition discriminability [d']), whereas the MCI cohort completed the CVLT-short form (CVLT-SF: total immediate recall, total long [10 minute] delay free recall, recognition discriminability [d']); visual episodic memory was quantified via delayed (10 minute) free recall of a complex figure (modified Benson figure); executive functions were quantified via a composite of digit span backward, modified Trail Making Test, Stroop Inhibition, lexical fluency (number of D-words/60″), and design fluency (DKEFS Condition 1); and language was quantified via a composite of the animal fluency task (number of animals/60″) and the 15-item Boston Naming Test.

MRI Processing

Before any prepossessing of the images, all T1-weighted images were visually inspected for quality control. Images with excessive motion or image artifact were excluded. T1-weighted images underwent bias field correction using N3 algorithm, and segmentation was performed using SPM12 (Wellcome Trust Center for Neuroimaging, London, UK, http://www.fil.ion.ucl.ac.uk/spm). An intra-subject template was created by non-linear diffeomorphic and rigid-body registration proposed by the symmetric diffeomorphic registration for longitudinal MRI framework. The intra-subject template was segmented also using SPM12's unified segmentation. A within-subject modulation was applied by multiplying the timepoints' jacobian with the intra-subject averaged tissues. A customized group template was generated from the within-subject average gray and white matter tissues and cerebrospinal fluid by non-linear registration template generation using it Large Deformation Diffeomorphic Metric Mapping framework. Modulated intra-subject gray and white matter were geometrically normalized to the group template and then smoothed~(8~mm full width half maximum Gaussian kernel) in the group template. Every step of the transformation was carefully inspected from the native space to the group template. For statistical purposes, linear and non-linear transformations between the group template space and International Consortium of Brain Mapping (ICBM) were applied.

© 2025 VandeVrede L et al. *JAMA Neurology*.

**Clinical syndrome**	AD syndrome	FTLD syndrome	AD syndrome						FTLD syndrome						
**Clinical diagnosis**			CN	MCI	LOAD	EOAD	lvPPA	PCA	CBS	PSP-RS	nfvPPA	svPPA	bvFTD	ALS	DLB
**n**	125	198	16	19	35	36	16	19	35	40	21	21	76	5	10
AD	108(86.4)	16(8.1)	5(31.2)	12(63.2)	30(85.7)	34(94.4)	16(100)	16(84.2)	8(22.9)	0(0)	2(9.5)	2(9.5)	4(5.3)	0(0)	3(30)
FTLD-tau	6(4.8)	90(45.5)	2(12.5)	4(21.1)	2(5.7)	0(0)	0(0)	0(0)	18(51.4)	27(67.5)	14(66.7)	2(9.5)	29(38.2)	0(0)	0(0)
FTLD-tau with AD	2(1.6)	24(12.1)	0(0)	0(0)	1(2.9)	0(0)	0(0)	1(5.3)	4(11.4)	10(25)	4(19)	0(0)	6(7.9)	0(0)	0(0)
FTLD-TDP	3(2.4)	54(27.3)	0(0)	2(10.5)	0(0)	1(2.8)	0(0)	0(0)	3(8.6)	0(0)	1(4.8)	15(71.4)	30(39.5)	5(100)	0(0)
FTLD-TDP with AD	2(1.6)	3(1.5)	0(0)	0(0)	1(2.9)	1(2.8)	0(0)	0(0)	1(2.9)	0(0)	0(0)	2(9.5)	0(0)	0(0)	0(0)
LBD	0(0)	2(1.0)	0(0)	0(0)	0(0)	0(0)	0(0)	0(0)	0(0)	2(5)	0(0)	0(0)	0(0)	0(0)	3(30)
LBD with AD	3(2.4)	1(0.5)	0(0)	0(0)	1(2.9)	0(0)	0(0)	2(10.5)	0(0)	1(2.5)	0(0)	0(0)	0(0)	0(0)	4(40)
Other	1(0.8)	7(3.5)	1(6.2)	1(5.3)	0(0)	0(0)	0(0)	0(0)	0(0)	0(0)	0(0)	0(0)	7(9.2)	0(0)	0(0)
Other with AD	0(0)	1(0.5)	0(0)	0(0)	0(0)	0(0)	0(0)	0(0)	1(2.9)	0(0)	0(0)	0(0)	0(0)	0(0)	0(0)
None	0(0)	0(0)	8(50)	0(0)	0(0)	0(0)	0(0)	0(0)	0(0)	0(0)	0(0)	0(0)	0(0)	0(0)	0(0)

eTable 1. Neuropathological distribution of primary pathology and AD co-pathology in each clinical syndrome. AD co-pathology was defined as intermediate or high AD neuropathological changes. Data is displayed a N (%).

© 2025 VandeVrede L et al. *JAMA Neurology*.

	P-tau217 CV, %	NfL CV, %	GFAP CV, %	Age Sample, y
**AD-related**	3.32±3.94 (0.99-4.25)	4.39±3.58 (1.96-6.18)	4.59±3.59 (1.99-6.30)	8.4±2.9 (6.2-11.07)
**FTLD-related**	4.79±3.89 (1.91-6.69)	3.23±2.56 (1.25-4.52)	5.05±4.12 (1.69-6.80)	7.9±2.9 (5.8-10.7)
**CU**	3.23±2.72 (0.79-4.44)	4.15±2.36 (1.17-7.25)	5.25±4.40 (1.63-8.32)	6.8±2.4 (4.6-8.4)
**MCI**	3.93±4.39 (1.52-4.27)	4.67±4.29 (1.93-5.37)	4.65±4.30 (1.62-6.40)	7.0±2.7 (4.6-10.1)
**LOAD**	2.94±2.15 (1.17-4.26)	4.03±3.62 (2.27-4.67)	4.32±3.29 (1.79-6.08)	8.7±2.9 (6.7-11.4)
**EOAD**	3.12±3.10 (0.65-4.24)	4.68±3.13 (2.40-7.43)	3.86±3.13 (1.90-4.37)	9.0±3.2 (6.5-12.0)
**lvPPA**	2.92±2.19 (1.02-4.19)	3.77±4.53 (1.09-4.29)	5.56±4.23 (2.62-8.54)	7.6±2.9 (5.2-9.1)
**PCA**	4.87±8.04 (1.93-4.58)	3.94±2.89 (1.80-5.93)	3.25±1.97 (1.70-4.84)	7.5±2.3 (6.0-7.9)
**CBS**	5.04±3.89 (1.93-7.31)	3.48±2.23 (1.74-5.16)	4.22±3.13 (1.36-6.00)	8.2±3.2 (6.3-11.1)
**PSP-RS**	5.09±3.82 (2.65-7.49)	3.78±3.50 (1.47-4.92)	4.95±3.09 (1.77-6.78)	7.7±2.7 (6.4-9.3)
**nfvPPA**	5.61±5.98 (0.89-8.08)	2.26±1.91 (0.92-3.05)	4.42±4.11 (1.27-5.87)	8.3±2.7 (5.9-11.2)
**svPPA**	4.86±3.28 (1.95-7.29)	3.57-2.35 (1.28-5.00)	4.59±3.52 (1.71-6.30)	9.0±2.6 (7.1-11.3)
**bvFTD**	4.28±3.59 (1.79-6.14)	3.06±2.40 (1.26-4.03)	5.78±4.69 (2.02-8.68)	7.8±3.2 (5.6-10.8)
**ALS**	7.18±1.09 (7.36-7.57)	2.17±2.75 (0.98-1.10)	6.74±4.50 (4.69-8.52)	9.3±2.7 (8.0-11.2)
**DLB**	5.65±2.65 (4.04-6.88)	4.74±3.66 (1.23-6.99)	6.17±5.18 (2.35-10.0)	7.5±2.3 (5.2-9.3)
**Total**	4.29±4.00 (1.64-6.00)	3.68±3.08 (1.41-4.90)	4.83±3.85 (1.80-6.59)	8.0±2.9 (5.8-10.7)

eTable 2. Technical performance in plasma biomarker assays. Coefficient of variation (CV) and age of sample within and across cohorts, displayed as mean±SD (IQR). Abbreviations: AD, Alzheimer's disease; ALS, amyotrophic lateral sclerosis; bvFTD, behavioral variant frontotemporal dementia; CBS, corticobasal syndrome; CU, cognitively unimpaired; DLB, dementia with Lewy bodies; EOAD, early-onset Alzheimer's disease; FTLD, frontotemporal lobar degeneration; GFAP, glial fibrillary acidic protein; LOAD, late-onset Alzheimer's disease; lvPPA, logopenic variant primary progressive aphasia; MCI, mild cognitive impairment; NfL, neurofilament light chain; nfvPPA, non-fluent variant primary progressive aphasia; PCA, posterior cortical atrophy; PSP-RS, progressive supranuclear palsy-Richardson's syndrome; p-tau217, phosphorylated tau 217; svPPA, semantic variant primary progressive aphasia.

© 2025 VandeVrede L et al. *JAMA Neurology*.

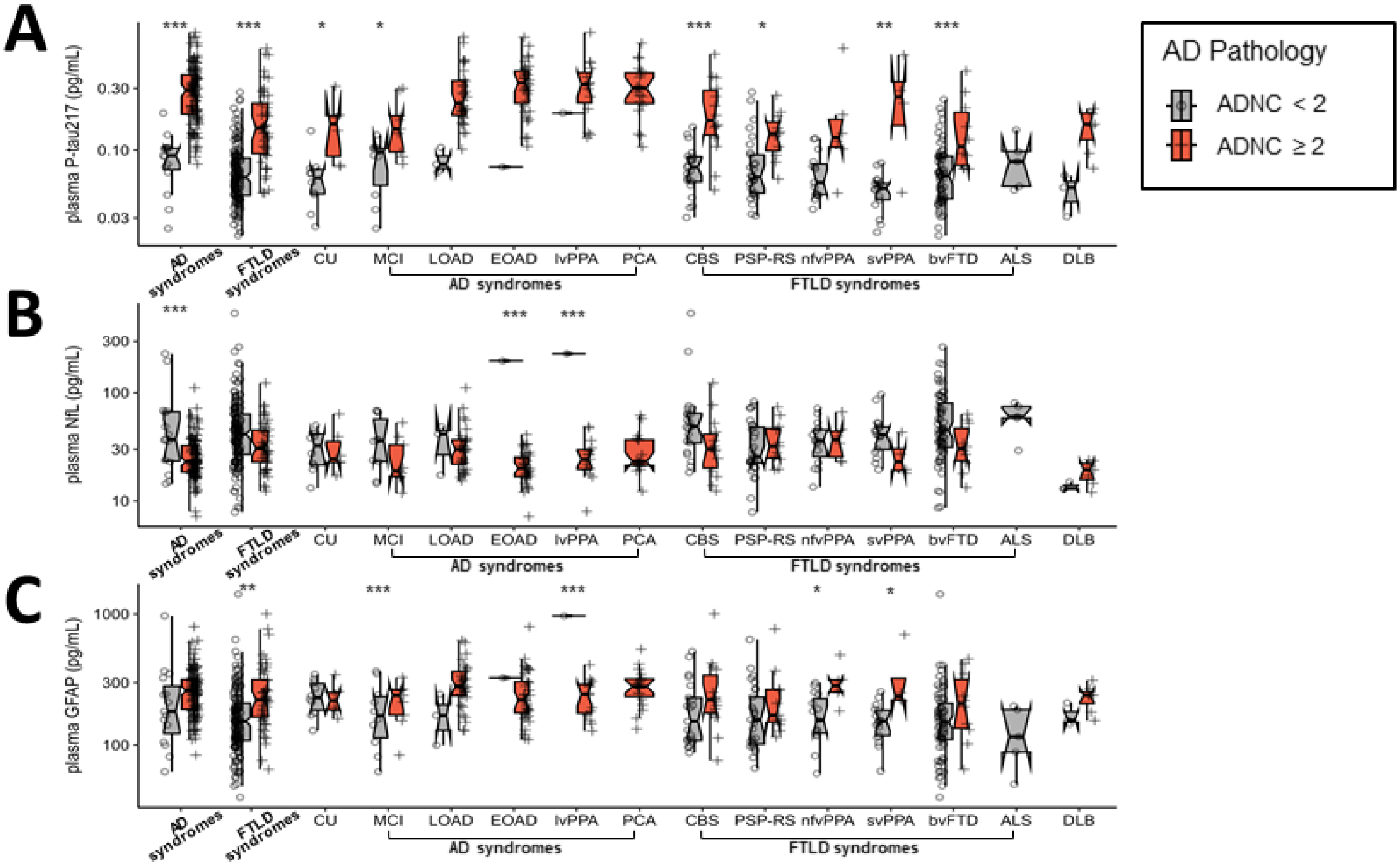

eFigure 1. Plasma Biomarkers Across Clinical Neurodegenerative Syndromes by Presence/Absence of AD Neuropathology. Plasma concentrations of (A) p-tau217, (B) NfL, and (C) GFAP are shown for each clinical neurodegenerative syndrome. The comparison between CU group and each clinical syndrome is denoted with statistical significance markers with covariates including age, sex, and the time interval between blood sampling and autopsy; *P<0.05, **P<0.01, ***P<0.001. Color represents the primary pathology. Shapes reflect the presence or absence of AD pathology.

© 2025 VandeVrede L et al. *JAMA Neurology*.

	P-tau217			NfL			GFAP		
	Full Cohort	AD-Related	FTLD-Related	Full Cohort	AD-Related	FTLD-Related	Full Cohort	AD-Related	FTLD-Related
ADNC > not	0.744**		0.637	0.641*		0.636	0.643		0.632
ADNC > low	0.947**		0.885**	0.731**		0.746	0.745**		0.755*
ADNC > int	0.965**	0.974*	0.956**	0.743**	0.931*	0.764	0.772**	0.768	0.788*
Thal > 0	0.748**		0.644	0.656*		0.64	0.657		0.64
Thal > 1	0.841**		0.723**	0.672*		0.666	0.676*		0.67
Thal > 2	0.938**		0.883**	0.7**		0.739	0.747**		0.771*
Thal > 3	0.953**		0.941**	0.723**		0.782	0.779**		0.832**
Thal > 4	0.948**	0.845*	0.928**	0.738**	0.773*	0.723	0.757**	0.728	0.772*
Braak > 0	0.897*		0.833	0.842		0.823	0.859		0.828
Braak > 1	0.876**		0.803*	0.757*		0.779	0.769*		0.785
Braak > 2	0.899**		0.806**	0.74**		0.718	0.727**		0.723
Braak > 3	0.922**		0.85**	0.741**		0.749*	0.754**		0.771*
Braak > 4	0.966**	1	0.956**	0.738**	0.908*	0.736	0.78**	0.88*	0.771*
Braak > 5	0.964**	0.951**	0.95**	0.742**	0.848*	0.695	0.776**	0.843	0.757*
CERAD > none	0.876**		0.776**	0.678*		0.707	0.711**		0.713
CERAD > sparse	0.905**		0.82**	0.671*		0.722	0.728**		0.728*
CERAD > mod	0.923**		0.85**	0.69**		0.729	0.762**		0.746*

AUC
< 0.75
0.75-0.79
0.80-0.84
0.85-0.89
0.90-0.94
0.95-1.00

eFigure 2. Diagnostic Performance of Plasma Biomarkers to Detect AD-related Neuropathology. Area under the curve (AUC) estimates from receiver operating curve characteristic (ROC) curves to predict neuropathology. Models included neuropathology as a binarized outcome with a plasma biomarker predictor. All models covaried for age, sex, and interval-to-autopsy. Pathology included Alzheimer's Disease Neuropathological Change (ADNC), Thal staging, Braak Staging, and Consortium to Establish a Registry for Alzheimer's Disease neuritic plaques (CERAD). Color represents heatmap of AUC. * p<0.05, ** p<0.01.

© 2025 VandeVrede L et al. *JAMA Neurology*.

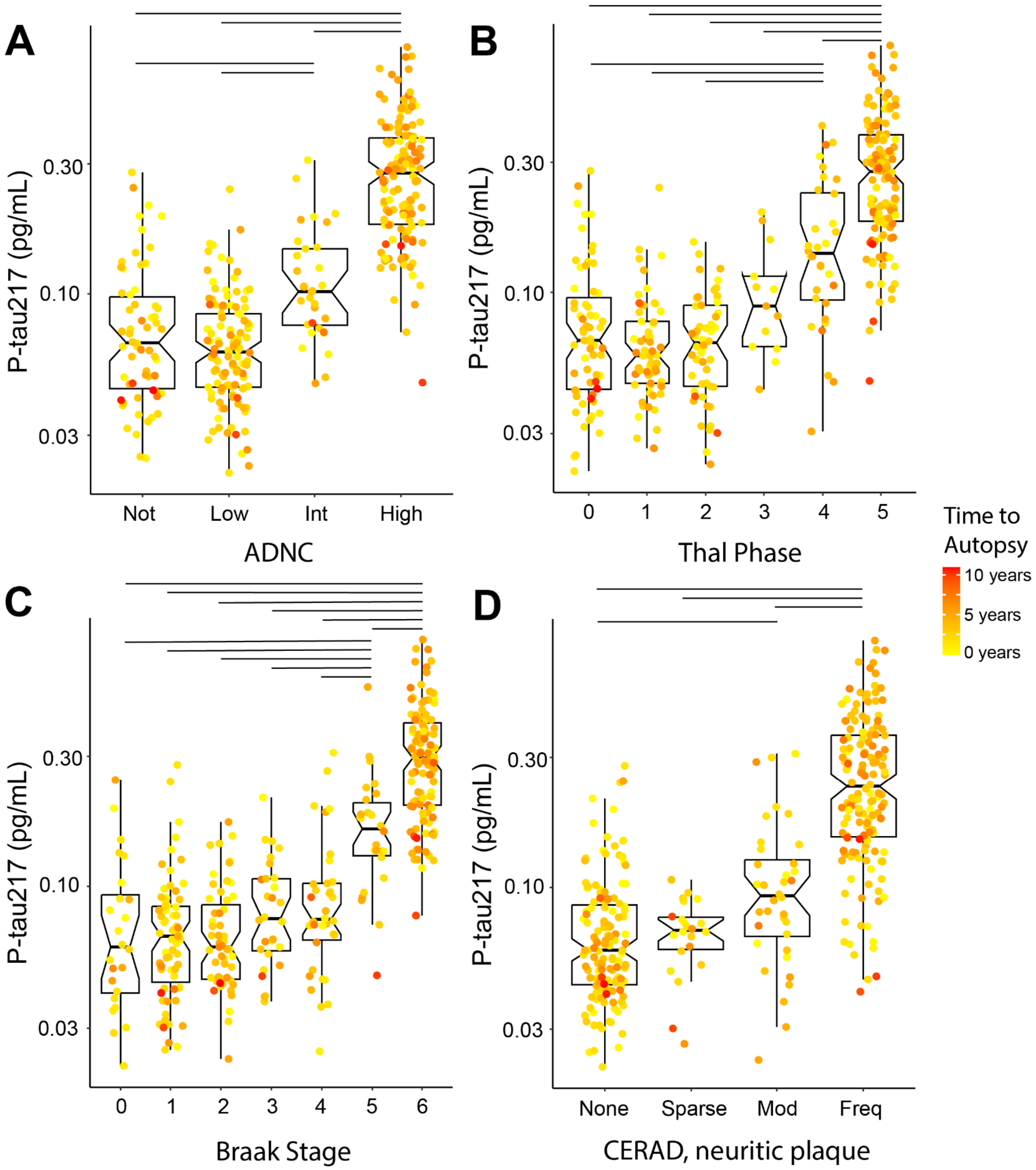

eFigure 3. Plasma concentrations of p-tau217 across neuropathological stages. Plasma p-tau217 concentrations across (A) ADNC, (B) Thal phases, (C) Braak stages, and (D) CERAD neuritic plaque scores are shown, color coded by time to

© 2025 VandeVrede L et al. *JAMA Neurology*.

autopsy. Statistical comparisons use post-hoc Dunn test with Bonferroni correction, with significance denoted by solid line between group.

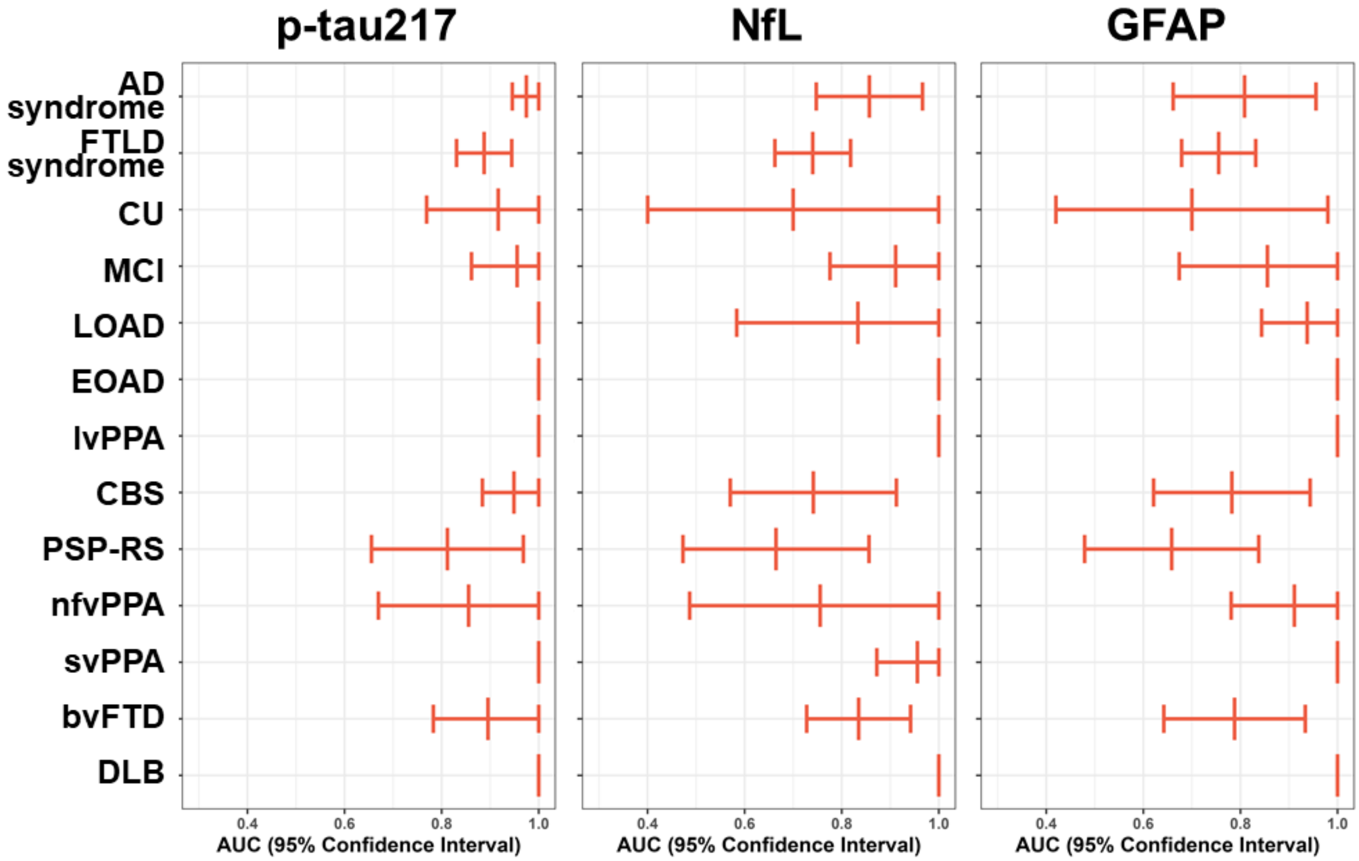

eFigure 4. Diagnostic performance of plasma biomarkers for AD neuropathology across clinical neurodegenerative syndromes. Forest plots show the area under the curve (AUC) with 95% confidence interval (CI) of plasma biomarkers: (A) P-tau217, (B) NfL, (C) GFAP in each clinical neurodegenerative syndromes to discriminate AD neuropathology. Note that PCA and ALS are not included due to neuropathological homogeneity.

© 2025 VandeVrede L et al. *JAMA Neurology*.

© 2025 VandeVrede L et al. *JAMA Neurology*.

eTable 3. Diagnostic performance of plasma biomarkers for AD neuropathology across clinical diagnosis.

**Clinical syndrome**	AD-related syndrome	FTLD-related syndrome		AD-related syndrome					FTLD-related syndrome						
**Clinical diagnosis**			CN	MCI	LOAD	EOAD	lvPPA	PCA	CBS	PSP-RS	nfvPPA	svPPA	bvFTD	ALS	DLB
**n**	125	198	16	19	35	36	16	19	35	40	21	21	76	5	10
**P-tau217**															
AUC	0.975	0.888	0.917	0.956	1.000	1.000	1.000	N/A	0.949	0.812	0.856	1.000	0.896	N/A	1.000
(95% CI)	(0.946-1.000)	(0.831-0.944)	(0.769-1.000)	(0.861-1.000)	(1.000-1.000)	(1.000-1.000)	(1.000-1.000)		(0.884-1.000)	(0.655-0.968)	(0.670-1.000)	(1.000-1.000)	(0.783-1.000)		(1.000-1.000)
Sensitivity	0.973	0.467	0.667	0.889	1.000	1.000	1.000	1.000	0.714	0.182	0.500	1.000	0.500	N/A	1.000
Specificity	0.800	0.961	0.900	0.900	1.000	1.000	1.000	N/A	0.952	0.931	0.933	1.000	0.970	1.000	1.000
True Positive	107	21	4	8	32	35	15	19	10	2	3	4	5	0	7
True Negative	12	147	9	9	3	1	1	0	20	27	14	17	64	5	3
False Positive	3	6	1	1	0	0	0	0	1	2	1	0	2	0	0
False Negative	3	24	2	1	0	0	0	0	4	9	3	0	5	0	0
Positive Predictive Value	0.973	0.778	0.800	0.889	1.000	1.000	1.000	1.000	0.909	0.500	0.750	1.000	0.714	N/A	1.000
Negative Predictive Value	0.800	0.860	0.818	0.900	1.000	1.000	1.000	N/A	0.833	0.750	0.824	1.000	0.928	1.000	1.000
Accuracy	0.931	0.842	0.813	0.944	1.000	1.000	1.000	1.000	0.882	0.725	0.850	1.000	0.935	1.000	1.000
**NfL**															
AUC	0.857	0.740	0.700	0.911	0.833	1.000	1.000	N/A	0.742	0.665	0.756	0.956	0.835	N/A	1.000
(95% CI)	(0.748-0.966)	(0.662-0.740)	(0.400-1.000)	(0.776-1.000)	(0.584-1.000)	(1.000-1.000)	(1.000-1.000)		(0.570-0.913)	(0.473-0.856)	(0.487-1.000)	(0.872-1.000)	(0.728-0.942)		(1.000-1.000)
Sensitivity	0.991	0.089	0.500	0.889	1.000	1.000	1.000	1.000	0.357	0.000	0.500	0.750	0.000	N/A	1.000
Specificity	0.267	0.974	0.800	0.800	0.000	1.000	1.000	N/A	0.857	0.966	0.933	0.882	0.985	1.000	1.000
True Positive	109	4	3	8	32	35	15	19	5	0	3	3	0	0	7
True Negative	4	149	8	8	0	1	1	0	18	28	14	15	65	5	3
False Positive	11	4	2	2	3	0	0	0	3	1	1	2	1	0	0
False Negative	1	41	3	1	0	0	0	0	9	11	3	1	10	0	0
Positive Predictive Value	0.908	0.500	0.600	0.800	0.914	1.000	1.000	1.000	0.625	0.000	0.750	0.600	0.000	N/A	1.000
Negative Predictive Value	0.800	0.784	0.727	0.889	N/A	1.000	1.000	N/A	0.667	0.718	0.824	0.938	0.867	1.000	1.000
Accuracy	0.883	0.750	0.733	0.842	0.914	1.000	1.000	1.000	0.767	0.700	0.810	0.818	0.867	1.000	1.000
**GFAP**															
AUC	0.809	0.755	0.700	0.856	0.938	1.000	1.000	N/A	0.782	0.658	0.911	1.000	0.788	N/A	1.000
(95% CI)	(0.661-0.956)	(0.679-0.831)	(0.420-0.980)	(0.674-1.000)	(0.844-1.000)	(1.000-1.000)	(1.000-1.000)		(0.621-0.944)	(0.479-0.838)	(0.781-1.000)	(1.000-1.000)	(0.642-0.934)		(1.000-1.000)
Sensitivity	1.000	0.289	0.667	0.889	1.000	1.000	1.000	1.000	0.571	0.182	0.667	1.000	0.300	N/A	1.000
Specificity	0.000	0.876	0.800	0.800	0.333	1.000	1.000	N/A	0.810	0.862	0.867	1.000	0.955	1.000	1.000
True Positive	110	13	4	8	32	35	15	19	8	2	4	4	3	0	7
True Negative	0	134	8	8	1	1	1	0	17	25	13	17	63	5	3
False Positive	15	19	2	2	2	0	0	0	4	4	2	0	3	0	0
False Negative	0	32	2	1	0	0	0	0	6	9	2	0	7	0	0
Positive Predictive Value	0.880	0.406	0.667	0.800	0.941	1.000	1.000	1.000	0.667	0.333	0.667	1.000	0.500	N/A	0.700
Negative Predictive Value	N/A	0.807	0.800	0.889	1.000	1.000	1.000	N/A	0.739	0.735	0.867	1.000	0.900	1.000	1.000
Accuracy	0.880	0.727	0.800	0.889	0.971	1.000	1.000	1.000	0.833	0.735	0.850	1.000	0.900	1.000	1.000

© 2025 VandeVrede L et al. *JAMA Neurology*.

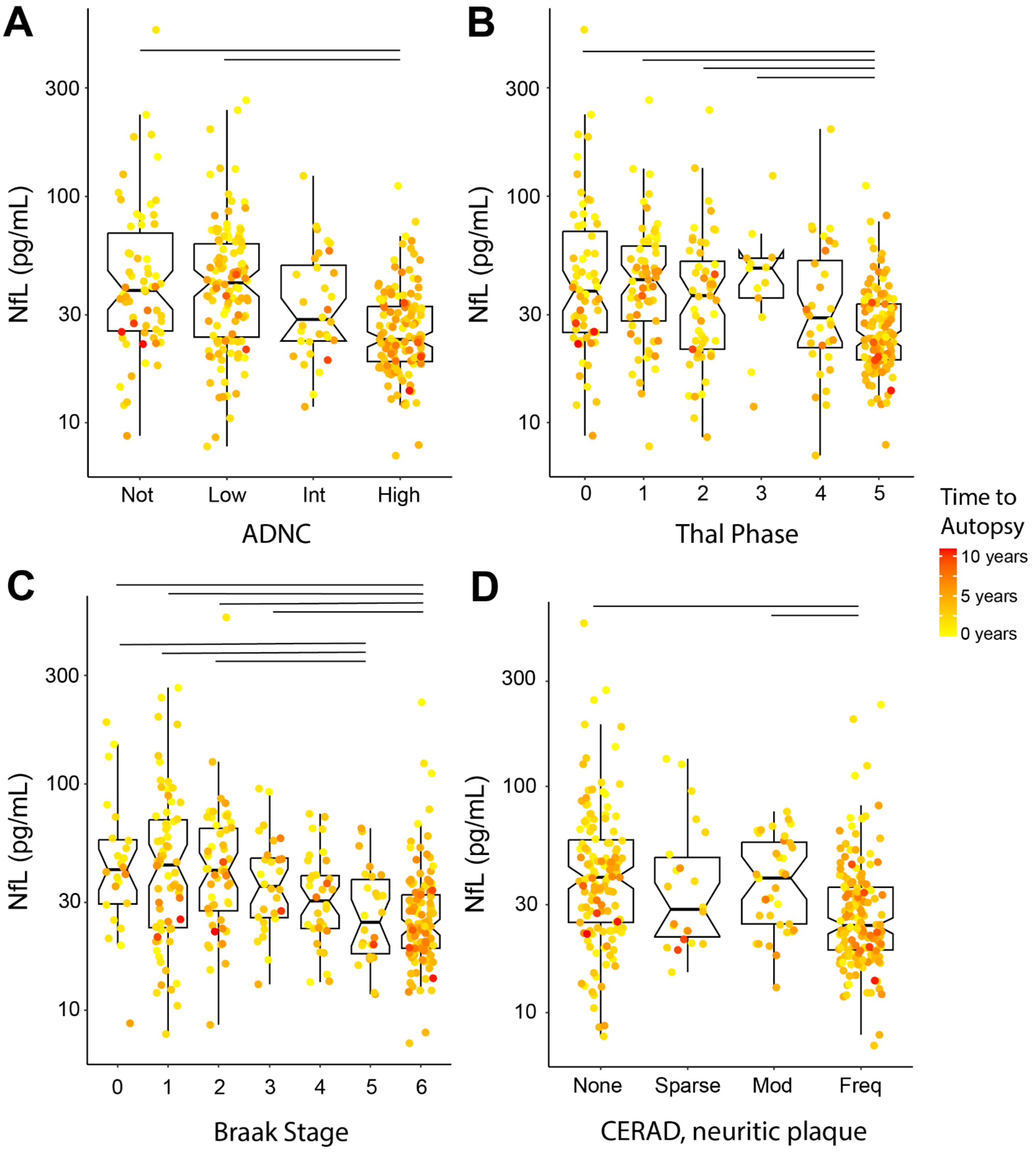

eFigure5. Plasma concentrations of NfL across neuropathological stages. Plasma NfL concentrations across (A) ADNC, (B) Thal phases, (C) Braak stages, and (D) CERAD neuritic plaque scores are shown, color coded by time to

© 2025 VandeVrede L et al. *JAMA Neurology*.

autopsy. Statistical comparisons use post-hoc Dunn test with Bonferroni correction, with significance denoted by solid line between group.

© 2025 VandeVrede L et al. *JAMA Neurology*.

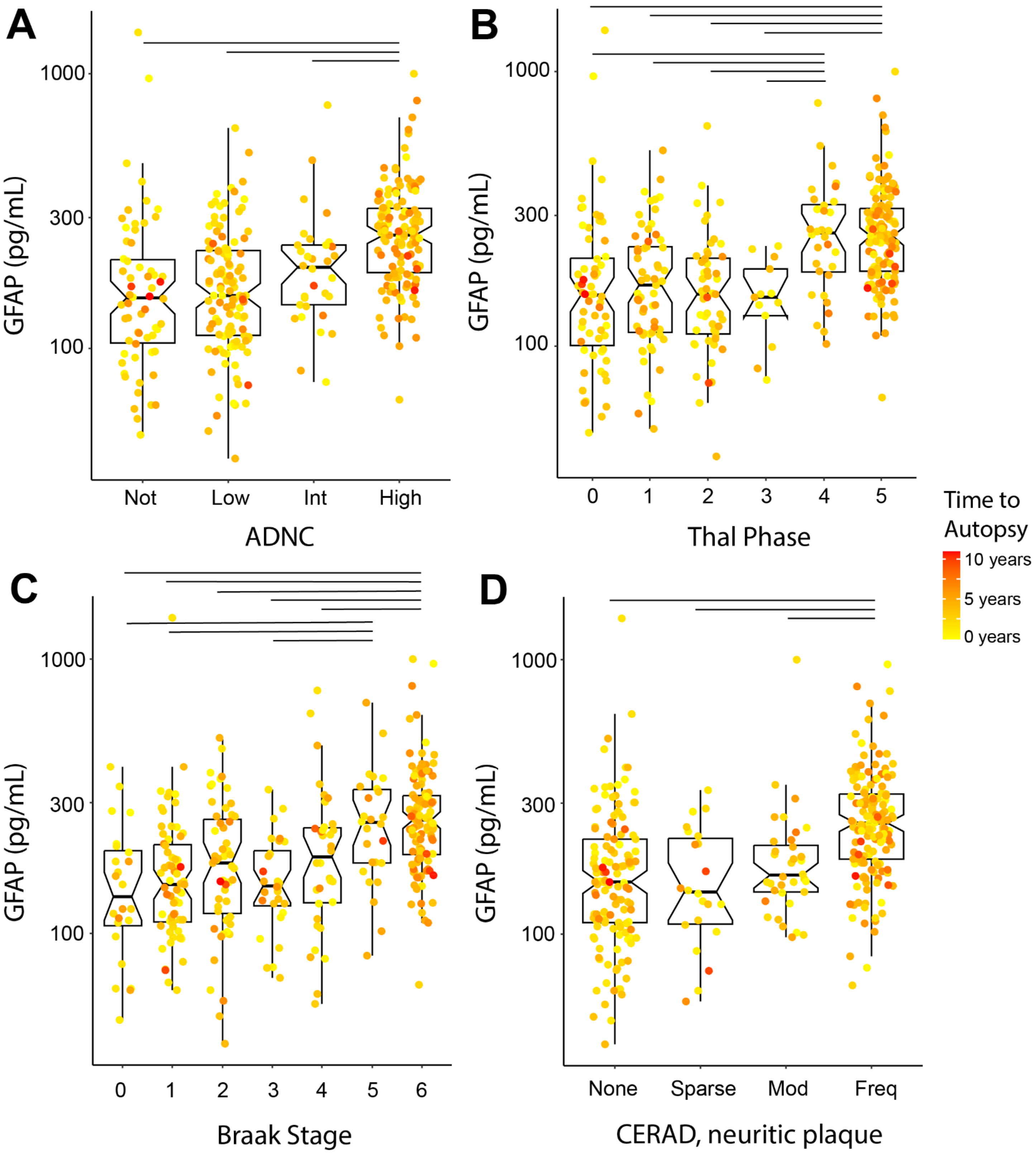

eFigure 6. Plasma concentrations of GFAP across neuropathological stages. Plasma GFAP concentrations across (A) ADNC, (B) Thal phases, (C) Braak stages, and (D) CERAD neuritic plaque scores are shown, color coded by time to autopsy. Statistical comparisons use post-hoc Dunn test with Bonferroni correction, with significance denoted by solid line between group.

© 2025 VandeVrede L et al. *JAMA Neurology*.

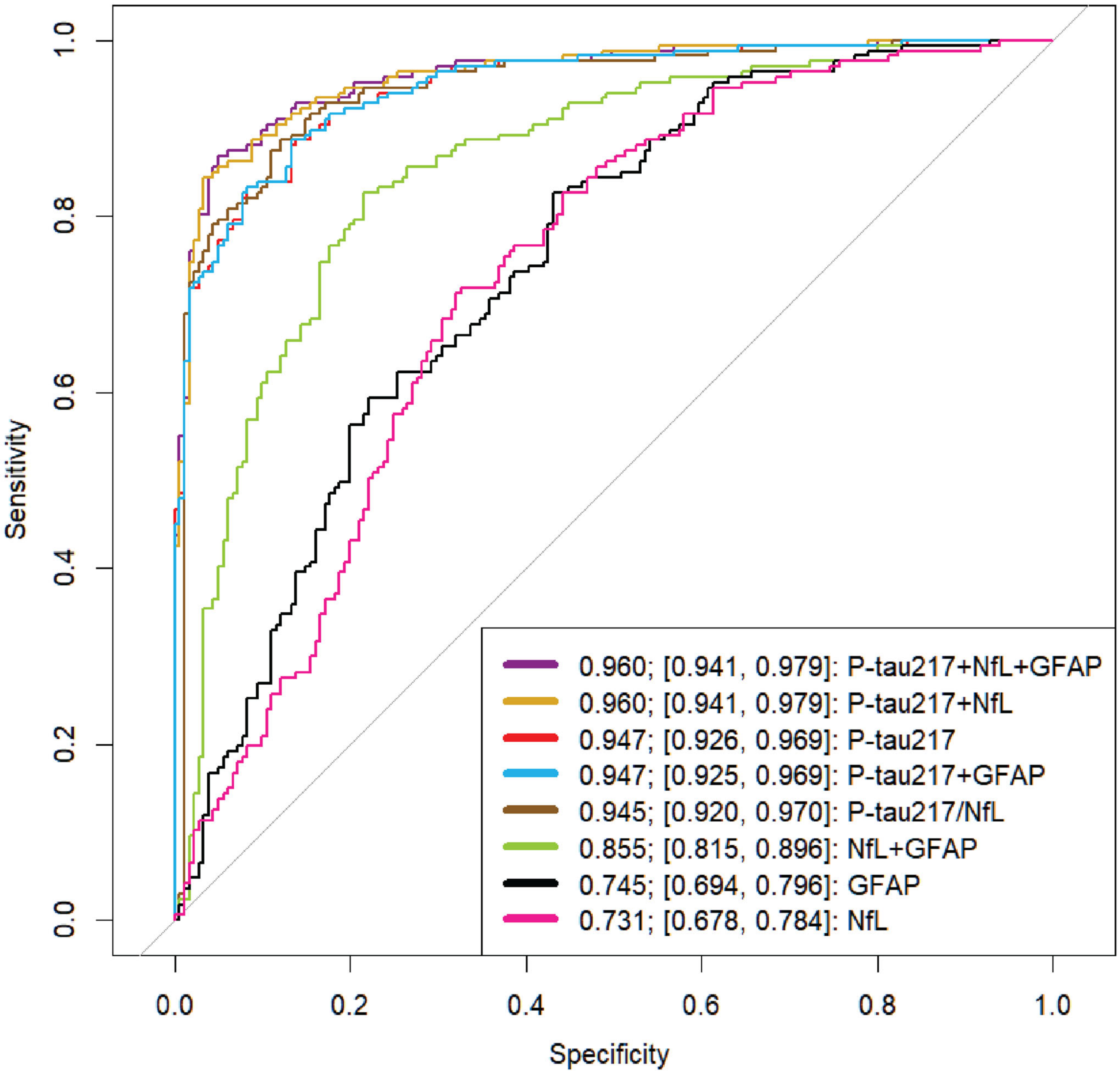

eFigure 7. Diagnostic Value of Plasma Biomarker Combinations to Detect AD Neuropathology. ROC curves are plotted for plasma p-tau217, GFAP, and NfL (and all combinations) to detect ADNC intermediate or higher within the entire cohort (N=349). Age, sex, and interval-to-autopsy were included as covariates. Inset indicates AUC and 95% CI. Matrix of Delong testing to compare ROC curves is included in **eTable 3**.

© 2025 VandeVrede L et al. *JAMA Neurology*.

Matrix of Delong tests for comparison of covariate-adjusted ROC models

	P-tau217+NfL+GFAP	P-tau217+NfL	P-tau217	P-tau217+GFAP	P-tau217/NfL	NfL+GFAP	GFAP	NfL
**P-tau217+NfL+GFAP**	---	NA	NA	NA	NA	P-tau217+NfL+GFAP	P-tau217+NfL+GFAP	P-tau217+NfL+GFAP
**P-tau217+NfL**	0.646	---	NA	NA	NA	P-tau217+NfL	P-tau217+NfL	P-tau217+NfL
**P-tau217**	0.089	0.091	---	NA	NA	P-tau217	P-tau217	P-tau217
**P-tau217+GFAP**	0.088	0.089	0.853	---	NA	P-tau217+GFAP	P-tau217+GFAP	P-tau217+GFAP
**P-tau217/NfL**	0.646	1	0.091	0.089	---	P-tau217/NfL	P-tau217/NfL	P-tau217/NfL
**NfL+GFAP**	<.0001	<.0001	<.0001	<.0001	<.0001	---	NfL+GFAP	NfL+GFAP
**GFAP**	<.0001	<.0001	<.0001	<.0001	<.0001	<.0001	---	NA
**NfL**	<.0001	<.0001	<.0001	<.0001	<.0001	<.0001	0.672	---

eTable 4. DeLong Comparison Across Plasma Biomarker Combinations. Each cell represents a comparison between the models listed in the row and column. The numbers below the diagonal represent p-values. The test in the upper diagonal indicates which model provided a significantly greater area under the curve (AUC) value. "NA" is listed if the Delong test was non-significant at an alpha of 0.5.

© 2025 VandeVrede L et al. *JAMA Neurology*.

	MMSE, score	Memory, z-score	Executive, z-score	Language, z-score	Visuospatial, z-score
**Age**	0.17±0.05 (0.29)*	0.02±0.02 (0.11)	0.03±0.01 (0.23)*	0.03±0.02 (0.12)	0.03±0.02 (0.14)
**Sex**	-0.04±0.89 (0.00)	0.54±0.27 (0.17)*	0.12±0.16 (0.06)	-0.18±0.37 (-0.04)	-0.31±0.31 (-0.08)
**Education**	0.24±0.17 (0.11)	0.07±0.05 (0.12)	0.09±0.03 (0.22)*	0.04±0.07 (0.04)	0.10±0.06 (0.14)
**AD Pathology**	-2.90±1.09 (0.22)*	-0.64±0.32 (0.17)*	-0.74±0.19 (0.31)*	-0.26±0.46 (0.05)	-0.88±0.37 (0.21)*
**Intercept**	-5.46±7.45	-8.39±2.27	-8.77±0.19*	-6.07±3.23	-8.48±2.58*
**R-squared**	0.11	0.07	0.16	0.02	0.08
**N**	154	145	151	156	140

eTable 5. Comparison of Neuropsychological Testing in FTLD-related syndromes with and without AD neuropathology. Results display linear regression models with MMSE score or z-scored cognitive domains as outcomes, presence of AD pathology as predictor, controlling for age, sex, and years of education. Data are presented as coefficients ± standard error (β coefficient) with asterisk representing significant difference ($p<0.05$).

© 2025 VandeVrede L et al. *JAMA Neurology*.
