# Supplementary material for: Detection of Alzheimer Neuropathology in Alzheimer and Non-Alzheimer Clinical Syndromes With Blood-Based Biomarkers
Source: JAMA Neurol. 2025 Feb 10;82(4):344–54. doi: 10.1001/jamaneurol.2024.5017 (PMC11811866; doi:10.1001/jamaneurol.2024.5017)
Supplement: Supplement 2. — Data sharing statement [file jamaneurol-e245017-s002.pdf]

Data Sharing Statement

Data
Data available: Yes
Data types: Deidentified participant data
How to access data: https://ucsf.co1.qualtrics.com/jfe/form/SV_01EHhRABhqgPgO2
When available: With publication

Supporting Documents
Document types: None

Additional Information
Who can access the data: Qualified researchers.
Types of analyses: Any purpose.
Mechanisms of data availability: After review of proposal request by MAC committee.
